# The metabolic score for visceral fat and risk of peripheral arterial disease in hypertension patients: a prospective cohort study

**DOI:** 10.3389/fendo.2026.1764481

**Published:** 2026-02-05

**Authors:** Shiping Li, Congcong Yan, Meihui Wu, Tao Wang, Lingjuan Zhu, Chao Yu, Weifang Zhang, Wei Zhou, Huihui Bao, Xiaoshu Cheng

**Affiliations:** 1 Department of Pharmacy, the Second Affiliated Hospital of Nanchang University, Nanchang, China; 2 School of Pharmacy, Jiangxi Science and Technology Normal University, Nanchang, China; 3 Department of Cardiovascular Medicine, the Second Affiliated Hospital, Jiangxi Medical College, Nanchang University, Nanchang, China; 4 Center for Prevention and Treatment of Cardiovascular Diseases, The Second Affiliated Hospital of Nanchang University, Nanchang, China; 5 Jiangxi Provincial Cardiovascular Disease Clinical Medical Research Center, Nanchang, China

**Keywords:** cohort study, hypertension, METS-VF, obesity, peripheral arterial disease

## Abstract

**Background:**

The Metabolic Score for Visceral Fat (METS - VF), a novel metric for evaluating visceral adipose tissue, has been demonstrated to exhibit a significant correlation with an elevated cardiovascular risk. Nevertheless, its relationship with peripheral artery disease (PAD) remains ambiguous. Consequently, the present study aimed to explore the association between METS-VF and PAD.

**Methods and results:**

This prospective study was based on a Chinese H-type hypertension cohort, comprising 6,452 patients. The association between METS-VF and PAD was evaluated using the Cox proportional hazards regression analysis and the method of restricted cubic splines (RCS). During an median follow-up time of 3.9 years, 266 PAD events occurred. The mean age of all participants was 63.20 ± 8.38 years. In the fully adjusted model, each 1-unit increase of METS-VF raised the risk of PAD by 21.0% (HR = 1.21, 95%CI: 1.07, 1.37). There was a saturation effect of METS-VF with an inflection point of 8.63 on PAD. For METS-VF < 8.63, each unit increase was associated with a 69.0% higher risk of PAD (HR=1.69, 95%CI: 1.32–2.16), while for METS-VF ≥8.63, there was no significant association between them (HR=0.93, 95%CI: 0.73–1.19) (P for log-likelihood ratio test= 0.002). Subgroup analysis further showed a significant interaction between METS-VF and current smoking status (P for interaction<0.05), with a stronger association observed in non-smokers.

**Conclusion:**

METS-VF exhibited a saturation effect on PAD in hypertensive adults in China. Increased METS-VF was positively associated with a higher risk of PAD among hypertensive adults with METS-VF < 8.63, and this association was more pronounced in non-smokers.

## Introduction

Peripheral arterial disease (PAD) is a manifestation of systemic atherosclerosis, which can lead to intermittent claudication, rest pain, and in severe cases, tissue gangrene and even amputation (1). It is closely associated with coronary heart disease, atrial fibrillation, stroke, and mortality (2–4). The global prevalence of PAD has risen rapidly in recent years (5). Nevertheless, a large number of people fail to receive treatment, which due to the fact that early peripheral artery disease (PAD) is asymptomatic (6). Therefore, identifying novel and effective biomarkers for monitoring PAD is crucial.

Extensive research indicates that obesity constitutes a significant risk factor for the incidence and mortality of PAD and various cardiovascular diseases (7–10). However, the traditional body mass index (BMI) cannot distinguish fat distribution, presenting limitations in risk assessment (11). Visceral fat exhibits greater metabolic activity than subcutaneous fat. Moreover, it is more closely associated with cardiovascular disease risk (12, 13). The Visceral Fat Metabolic Score (METS-VF) is a novel indicator proposed by Bello-Chavolla et al. It integrates multiple factors including age, gender, waist-to-height ratio, and insulin resistance score. When assessing visceral fat, this composite metric demonstrates superior evaluative efficacy compared to conventional indicators (14). It has been confirmed to be closely related to diseases such as diabetes, hypertension, cardiovascular diseases, and cardiorenal metabolic syndrome (14–16). At present, there are few studies exploring the association between METS-VF and PAD in hypertensive patients. Hypertension is a major risk factor for PAD (17), with a prevalence rate already as high as 23% among adult residents in China (18). These patients often have higher overall lipid levels, and their cardiometabolic burden may be more pronounced compared to non-hypertensive individuals.

Consequently, the objective of this study is to utilize a prospective cohort to analyze the association between METS-VF and PAD among Chinese hypertensive patients.

## Methods

### Study population

The study participants were drawn from the China Hypertension Registry (Registration Number: ChiCTR1800017274). This observational study was designed to systematically investigate the prevalence, treatment status, and prognostic factors of hypertension in China by establishing a nationwide cohort of hypertensive patients. The inclusion criteria included (1): age ≥ 18 years (2); diagnosis of hypertension, defined as a resting systolic blood pressure (SBP) ≥ 140 mmHg, or a diastolic blood pressure (DBP) ≥ 90 mmHg, or a documented history of hypertension, or the use of antihypertensive medication at baseline (19) (3); provision of signed informed consent. The exclusion criteria included (1): presence of psychiatric or neurological dysfunction impairing the ability to express consent (2); inability to guarantee completion of follow-up as planned or planned relocation in the near future (3); not suitable for long-term follow-up upon assessment by the research physician.

**Figure 1:**
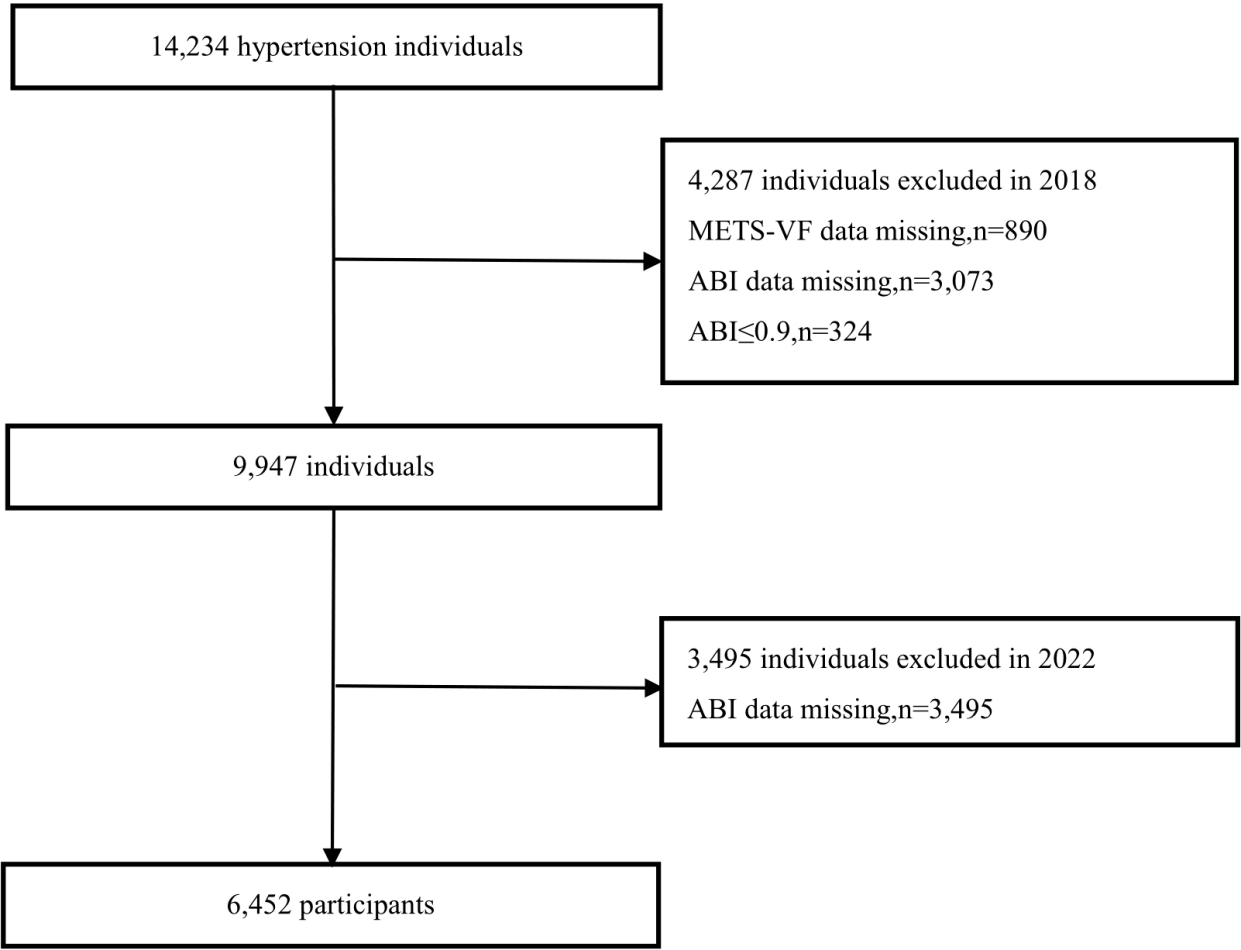
Flow chart of participants.

From March to August 2018, a total of 14,234 hypertensive participants at baseline were enrolled in Wuyuan County, the follow-up was completed from June to August in 2022. After excluding participants lost to follow-up (n=2), those with missing baseline data required for calculating METS-VF (n=890), those with missing PAD at baseline (n=3,073), those with pre-existing PAD at baseline (n=324), and those with missing PAD information at follow-up (n=3,495), a final total of 6,452 subjects were included in the analysis. The participant selection flowchart is presented in **Figure 1**.

This study was conducted in accordance with the principles of the Declaration of Helsinki. It was approved by the Biomedical Ethics Committee of Anhui Medical University (No. CH1059) and the Biomedical Ethics Committee of the Second Affiliated Hospital of Nanchang University (No. 2018019). All patients signed an informed consent before enrollment.

### Data collection

Baseline data were collected by uniformly trained staff through standardized questionnaires and health interviews. The collected demographic information included age, sex, lifestyle factors (smoking, alcohol consumption), medical history (diabetes, family history of coronary heart disease and hypertension), and medication use (antihypertensive and lipid-lowering drugs). Anthropometric measurements, including body weight, height, and waist circumference, were also recorded. Seated blood pressure was measured using an electronic sphygmomanometer (Omron; Dalian, China) after participants had rested quietly for a 5-min rest, with a 30-s interval between measurements. The average of the three measurements was used.

All participants were instructed to fast for at least 12 hours overnight prior to blood collection. Fasting venous blood samples were drawn the following morning. Following centralized collection and cryopreservation of all blood samples, these were uniformly transported to Shenzhen Biaojia Biotechnology Laboratory. Testing was conducted using fully automated biochemical analyzers from Beckman Coulter, USA, to measure fasting blood glucose (FPG), homocysteine (Hcy), triglycerides (TG), total cholesterol (TC), high-density lipoprotein cholesterol (HDL-C), low-density lipoprotein cholesterol (LDL-C), and indicators of liver function and kidney function, etc. The estimate glomerular filtration rate (eGFR) was calculated using the Chronic Kidney Disease Epidemiology Collaboration (CKD-EPI) equation (20).

### Definition of PAD

PAD was defined as an ankle-brachial index (ABI) ≤ 0.9 in either leg (21). Blood pressure in both lower limbs was measured using an Omron BP-203RPE III sphygmomanometer (Omron Health Care, Kyoto, Japan) after subjects rested supine for at least ten minutes. ABI for each lower limb was calculated as the ratio of ankle systolic pressure to brachial systolic pressure. ABI for each leg was calculated by dividing the measured ankle SBP by the brachial artery SBP. ABI was measured once at the baseline survey and once again during the follow-up survey.

### Calculation of METS-VF

METS-VF was determined by applying the following equations:


WHtR=WC(cm)/height(cm)

(22)


$$\begin{array}{c} \text{METS}-\text{IR }=\;\left(\text{Ln }\left(2\;\times \text{ FPG }\left(\text{mg}/\text{dL}\right)\right)\;+\text{ TG }\left(\text{mg}/\text{dL}\right)\right)\\ \;\times \text{ BMI }\left(\text{kg}/{\text{m}}^{2}\right)/\text{Ln }\left(\text{HDL}-\text{C}\left(\text{mg}/\text{dL}\right)\right) \end{array}$$

(23)


$$\begin{array}{c} \text{METS-VF }=\;4.466+0.011\times {\left(\text{Ln}\left(\text{METS}-\text{IR}\right)\right)}^{3}+\;3.239\\ \times \left(\text{Ln}\left(\text{WHtr}\right)\right)3\;+\;0.319\times \left(\text{Gender}\right)+0.594\times \left(\text{Ln}\left(\text{Age}\right)\right) \end{array}$$

(14)

### Statistical analysis

Baseline characteristics were described using mean ± standard deviation for continuous variables. Categorical variables were described as frequency (%). Study participants were categorized into three groups based on tertiles of METS-VF. When comparing indicators across these groups, we employed one-way analysis of variance for normally distributed variables and the Kruskal-Wallis test for those exhibiting non-normal distributions. Cox proportional hazard regression was used to evaluate hazard ratio (HR) and 95% confidence interval (CI) for the association between METS-VF and PAD. Three models were constructed: Model 1 was adjusted for age and sex; Model 2 was adjusted for age, sex and BMI; and Model 3 was adjusted for age, sex, BMI, DBP, SBP, diabetes, coronary heart disease (CHD), LDL-C, TC, eGFR, BMI, Hcy, use of antihypertensive drugs, and use of lipid-lowering drugs. Additionally, restricted cubic spline (RCS) analyses were employed to examine the dose-response relationship between METS-VF and PAD. Stratified analyses and interaction tests were conducted to identify potential effect modifiers influencing the association between METS-VF and PAD. The proportional hazards assumption was assessed using the Schoenfeld residuals. For the sensitivity analysis, we excluded BMI when conducting the Cox regression adjustment for covariates, and a Cox regression analysis was conducted for the BMI groups (<24 kg/m^2^ and ≥ 24 kg/m^2^).

All statistical analyses were performed using R software (http://www.r-project.org) and EmpowerStats (http://www.empowerstats.com). A two-sided p-value < 0.05 was considered statistically significant.

## Results

### Baseline characteristics of study participants

**Table 1** presents the baseline characteristics of participants stratified by METS-VF tertiles. The final analysis included 6,452 hypertensive patients with a mean (SD) age of 63.20 (8.38) years, of whom 2,936 (45.51%) were males. Compared to those in the lower METS-VF tertiles, participants in the highest tertile exhibited significantly higher levels of BMI, waist circumference (WC), FBG, DBP, TG, LDL-C, and uric acid (UA). Participants with higher METS-VF had a higher prevalence of stroke, diabetes, and usage of antihypertensive and lipid-lowering medications; had lower HDL-C levels and were less likely to be current smokers and drinkers.

**Table 1:** Baseline characteristics of subjects stratified according to tertiles of METS-VF.

Variables	Total	METS-VF			P value
		Q1(≤7.30)	Q2(≥7.31,<8.06)	Q3(≥8.06)	
N	6452	2137	2162	2153	
Male, n(%)	2936 (45.51)	1343 (62.85)	868 (40.15)	725 (33.67)	<0.001
Age, years	63.20 ± 8.38	64.01 ± 8.35	62.78 ± 8.47	62.81 ± 8.28	<0.001
BMI, kg/m^2^	23.73 ± 3.93	20.92 ± 2.33	24.12 ± 2.69	26.15 ± 4.45	<0.001
WC, cm	83.42 ± 9.62	75.24 ± 7.15	84.79 ± 6.81	90.16 ± 8.13	<0.001
SBP, mmHg	148.57 ± 17.31	148.45 ± 17.67	149.18 ± 17.24	148.09 ± 17.02	0.107
DBP, mmHg	89.31 ± 10.39	88.75 ± 10.62	89.74 ± 10.26	89.42 ± 10.28	0.006
Hcy, μmol/L	17.40 ± 9.98	17.99 ± 9.67	17.05 ± 9.85	17.16 ± 10.38	0.003
FPG, mmol/L	6.17 ± 1.53	5.91 ± 1.17	6.16 ± 1.38	6.44 ± 1.89	<0.001
TC, mmol/L	5.23 ± 1.08	5.28 ± 1.07	5.44 ± 1.07	4.97 ± 1.05	<0.001
TG, mmol/L	1.74 ± 1.16	1.24 ± 0.68	1.70 ± 0.91	2.28 ± 1.48	<0.001
HDL-C, mmol/L	1.64 ± 0.42	1.94 ± 0.42	1.67 ± 0.32	1.32 ± 0.23	<0.001
LDL-C, mmol/L	3.04 ± 0.81	2.88 ± 0.82	3.20 ± 0.81	3.02 ± 0.75	<0.001
UA, μmol/L	410.51 ± 119.16	405.69 ± 117.57	406.52 ± 118.92	419.32 ± 120.51	<0.001
eGFR, ml/min/1.73 m^2^	90.63 ± 18.69	89.95 ± 18.88	92.09 ± 18.01	89.85 ± 19.09	<0.001
Diabetes, n (%)	1166 (18.07)	233 (10.90)	369 (17.07)	564 (26.20)	<0.001
Stroke, n (%)	378 (5.86)	113 (5.29)	121 (5.60)	144 (6.69)	0.121
CHD, n (%)	294 (4.56)	85 (3.98)	91 (4.21)	118 (5.48)	0.039
Antihypertensive drugs, n (%)	4285 (66.42)	1333 (62.38)	1449 (67.02)	1503 (69.84)	<0.001
Lipoprotein-lowering drugs, n (%)	218 (3.38)	49 (2.29)	67 (3.10)	102 (4.74)	<0.001
Current smoking, n (%)	1616 (25.05)	767 (35.89)	437 (20.21)	412 (19.14)	<0.001
Current drinking, n (%)	1501 (23.27)	728 (34.07)	453 (20.95)	320 (14.87)	<0.001

Data are the mean± SD, median (range), or number (percentage).

BMI, body mass index; WC, Waist Circumference; SBP, systolic blood pressure; DBP, diastolic blood pressure; FPG, fasting plasma glucose; TC, total cholesterol; TG, triglycerides; HDL-C, high density lipoprotein cholesterol; LDL-C, low density lipoprotein cholesterol; UA, uric acid; eGFR, estimated glomerular filtration rate; Hcy, homocysteine; CHD, coronary heart disease.

### Association between METS-VF and PAD

**Table 2** presents a significant positive association was observed between METS-VF and PAD. In the fully adjusted model (Model 3), each 1-unit increment in METS-VF was associated with a 21% increased risk of PAD (HR = 1.21; 95% CI: 1.07, 1.37). Compared with participants in the lowest tertile (Q1), those in the second (Q2) and third (Q3) tertiles had significantly higher risks of PAD, with HRs of 1.57 (95% CI: 1.11, 2.21) and 2.30 (95% CI: 1.50, 3.51) (P for trend <0.001). The consistent associations were also revealed by the sensitivity analyses, including Cox regression analysis between METS-VF and PAD conducted with adjusting for covariates excluding BMI (**Supplementary Table S1**), and that conducted in two groups with BMI < 24 kg/m^2^ and BMI ≥ 24 kg/m^2^ (**Supplementary Table S2**).

**Table 2:** Association between MTES-VF and PAD in different models.

MTES-VF	N	Events, n (%)	PAD, HR (95%CI)		
			Model	Model 2	Model 3
Per 1 unit increase	6452	266 (4.12)	1.11 (1.00, 1.23)	1.24 (1.11, 1.38)	1.21 (1.07, 1.37)
Tertiles					
Q1(<7.30)	2137	87 (4.07)	1.0	1.0	1.0
Q2(≥7.31,<8.06)	2162	89 (4.12)	1.46 (1.06, 2.02)	1.61 (1.15, 2.26)	1.57 (1.11, 2.21)
Q3(≥8.06)	2153	90 (4.18)	1.83 (1.32, 2.52)	2.38 (1.61, 3.51)	2.30 (1.50, 3.51)
*P* for trend			0.016	<0.001	0.001

Model 1 was adjusted for age, sex.

Model 2 was adjusted for age, sex, BMI.

Model 3 was adjusted for age, sex, BMI, DBP, SBP, current smoking, CHD, LDL-C, TC, eGFR, hcy, current drinking, diabetes, antihypertensive drugs, lipoprotein-lowering drugs.

BMI, body mass index; SBP, systolic blood pressure; DBP, diastolic blood pressure; CHD, coronary heart disease; TC, total cholesterol; LDL-C, low density lipoprotein cholesterol; eGFR, estimated glomerular filtration rate; Hcy, homocysteine.

### Threshold effect of METS-VF on PAD

**Figure 2:**
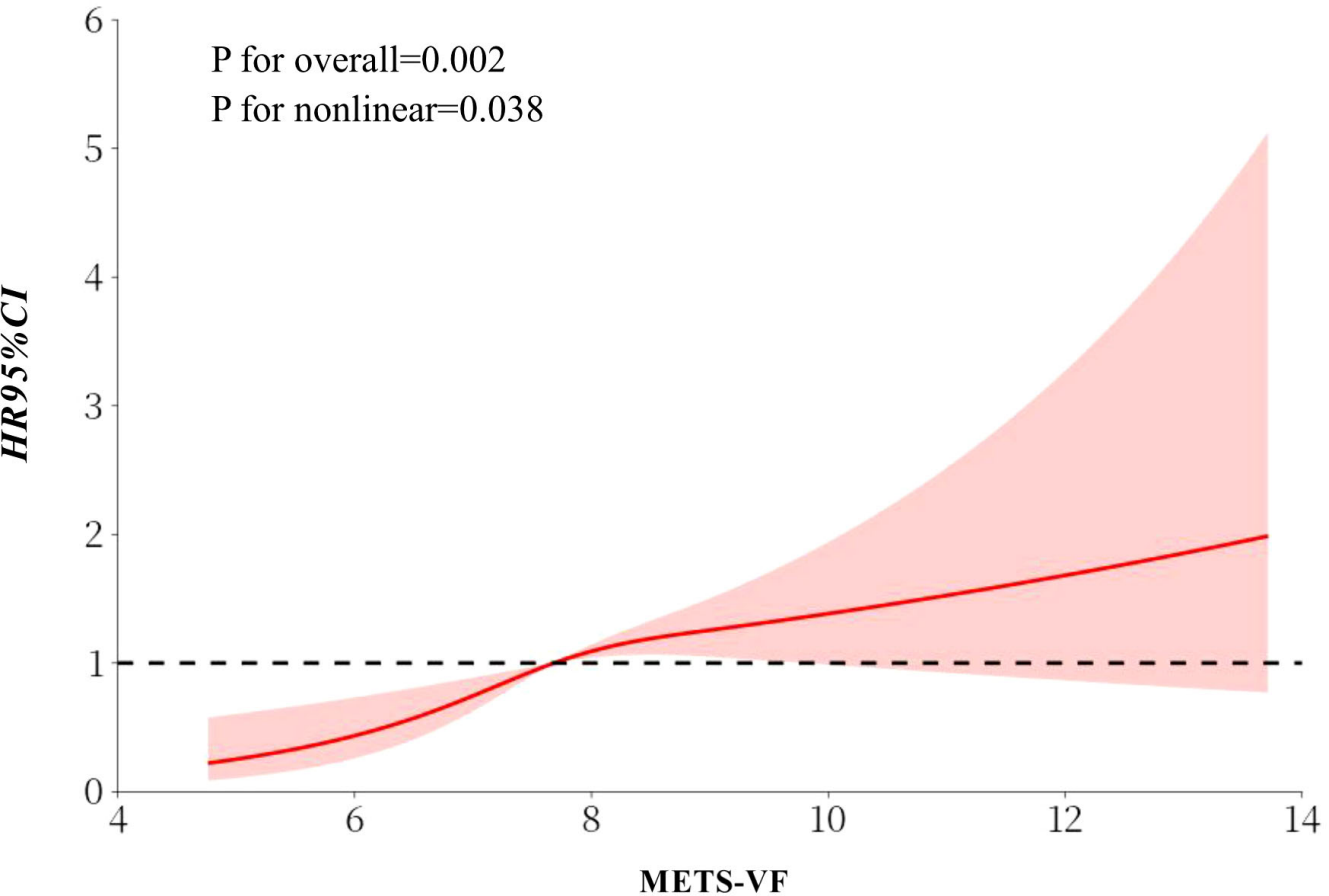
Dose-response relationship of METS-VF with PAD using restricted cubic splines. The model was adjusted for age, sex, BMI, DBP, SBP, Current smoking, CHD, LDL-C, TC, eGFR, Hcy, Current drinking, Diabetes, Antihypertensive drugs, Lipoprotein-lowering drugs. BMI, body mass index; SBP, systolic blood pressure; DBP, diastolic blood pressure; CHD, coronary heart disease; TC, total cholesterol; LDL-C, low density lipoprotein cholesterol; eGFR, estimated glomerular filtration rate; Hcy, homocysteine.

The restricted cubic spline (RCS) analysis performed based on Model 3 indicated a threshold effect of METS-VF on PAD (**Figure 2**). Using a two-piecewise Cox regression model, an inflection point was determined at a METS-VF value of 8.63. For METS-VF < 8.63, the adjusted HR (95% CI) was 1.69 (1.32, 2.16), and the adjusted HR (95% CI) was 0.93 (0.73, 1.19) for METS-VF ≥ 8.63 (P for log-likelihood ratio test = 0.002) (**Table 3**).

**Table 3:** Saturation effect analysis of METS-VF on PAD.

METS-VF	N	Events (%)	PAD, HR (95%CI)	P value
Per 1 unit increase	6452	266 (4.12)	1.21 (1.07, 1.37)	0.002
Inflection point				
<8.63	5459	226 (4.14)	1.69 (1.32, 2.16)	<0.001
≥8.63	993	40 (4.03)	0.93 (0.73, 1.19)	0.583
P for log likelihood ratio test			0.002	

Model was adjusted for age, sex, BMI, DBP, SBP, current smoking, CHD, LDL-C, TC, eGFR, hcy, current drinking, diabetes, antihypertensive drugs, lipoprotein-lowering drugs.

BMI, body mass index; SBP, systolic blood pressure; DBP, diastolic blood pressure; CHD, coronary heart disease; TC, total cholesterol; LDL-C, low density lipoprotein cholesterol; eGFR, estimated glomerular filtration rate; Hcy, homocysteine.

### Subgroup analysis

**Figure 3:**
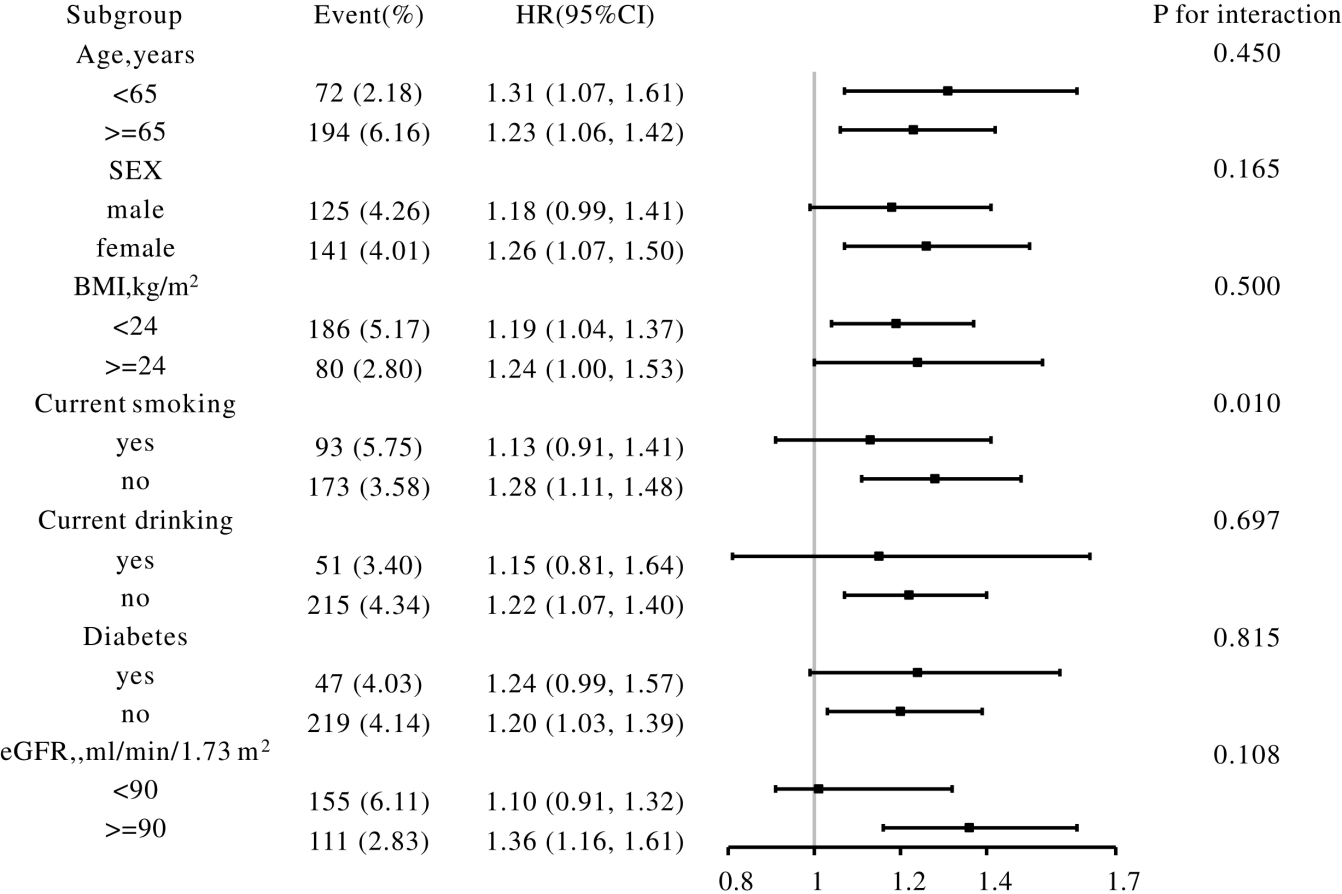
Subgroup analyses of the effect of METS-VF on PAD. Each subgroup analysis was adjusted, if not stratified, for age, sex, BMI, DBP, SBP, Current smoking, CHD, LDL-C, TC, eGFR, Hcy, Current drinking, Diabetes, Antihypertensive drugs, Lipoprotein-lowering drugs. BMI, body mass index; SBP, systolic blood pressure; DBP, diastolic blood pressure; CHD, coronary heart disease; TC, total cholesterol; LDL-C, low density lipoprotein cholesterol; eGFR, estimated glomerular filtration rate; Hcy, homocysteine.

The association between METS-VF and PAD was consistent across various subgroups, including those stratified by sex, age, BMI, eGFR, current smoking, current drinking, and diabetes. However, a test for interaction revealed that the strength of this association was significantly modified by smoking status (P for interaction<0.05), with a more pronounced effect among non-smokers (**Figure 3**).

## Discussion

This study is the first to investigate the association between METS-VF and PAD in Chinese patients with hypertension. Our results demonstrated a threshold effect of METS-VF on PAD, with an inflection point of METS-VF identified at 8.63. Furthermore, a more pronounced association observed in non-smokers.

Substantial evidence supports METS-VF as a robust cardiometabolic risk marker, with its predictive value for diverse cardiovascular outcomes well established. A study involving a Taiwanese health examination population found that, METS-VF remained significantly positively associated with the risk of coronary artery calcification, with an optimal predictive cut-off value of 6.405 (24). Zhang et al. demonstrated that METS-VF was significantly associated with heart failure risk, and participants in the highest quartile had a 2.731-fold higher risk compared to those in the lowest quartile (25). Xie et al. reported that among patients with non-alcoholic fatty liver disease, METS-VF was positively associated with both cardiovascular mortality and all-cause mortality (26). Elevated systolic blood pressure, a key feature of hypertension, is a well-established risk factor for PAD (27). However, the relationship between METS-VF and PAD has not been previously examined specifically in hypertensive populations. Our study is the first to demonstrate a positive association between METS-VF and PAD in this specific population.

Our research indicated that when METS-VF was ≥ 8.63, its association with PAD risk ceased to be statistically significant. This pattern was consistent with a prior study showing a similar loss of association for carotid atherosclerosis beyond a METS-VF of 8.09 (28). In our study, the high METS-VF group received more intensive lipid-lowering therapy and exhibited lower levels of TC, TG, HDL-C, and LDL-C during the follow-up survey. This effective delay in the progression of atherosclerosis may have mitigated the observable risk. Consequently, which suggests that this nonlinear pattern may have a certain degree of generalizability.

This study also confirmed an interaction between METS-VF and smoking status, with the association between METS-VF and PAD being more pronounced in non-smokers. Consistent with this, Tan et al. also reported a significant interaction between METS-VF and smoking status, reporting a stronger association between METS-VF and cardiovascular disease in non-smokers (29). This phenomenon may be related to smoking itself, which is an independent risk factor for PAD (30). Furthermore, related studies indicate that the population attributable fraction for cardiovascular diseases due to smoking is higher than that for BMI (31). This implies that in comparison with smoking, visceral adiposity might represent an even more critical modifiable risk factor for cardiovascular outcomes, thereby further highlighting the substantial and non-negligible risk posed by excess visceral fat.

The positive correlation between METS-VF and PAD can be explained by the impact of visceral fat on atherosclerosis. Visceral fat stimulates the secretion of pro-inflammatory factors (such as TNF-α and IL-6), and due to abnormal lipolysis leading to increased free fatty acids, these factors jointly induce systemic insulin resistance and chronic low-grade inflammation (32, 33). Insulin resistance impairs the protective IRS-1/PI3K/Akt signaling pathway, leading to endothelial nitric oxide synthase dysfunction and compromised vasodilation. Concurrently, compensatory hyperinsulinemia activates pro-atherogenic MAPK pathways, promoting vascular smooth muscle cell proliferation and inflammation (34, 35). These disorders collectively result in the classic atherosclerotic dyslipidemia with impaired apolipoprotein These dysregulations collectively result in classic atherosclerotic dyslipidemia coupled with impaired apolipoprotein B clearance—manifesting as elevated triglycerides, low HDL-C, and increased small dense LDL particles—further exacerbated by reduced secretion of protective adipokines such as adiponectin (36) (37),. These dysregulations collectively contribute to the classic atherosclerotic profile of dyslipidemia coupled with impaired apolipoprotein B clearance—manifesting as elevated triglycerides, low HDL-C, and increased small dense LDL particles—further exacerbated by reduced secretion of protective adipokines such as adiponectin (36, 37). This cascade establishes a vicious cycle that accelerates the development and progression of atherosclerosis, the fundamental pathological basis of PAD.

METS-VF estimates visceral fat using standard blood tests and simple body measurements. It has been shown to outperform traditional obesity indicators in predicting visceral fat content and conditions such as hypertension, diabetes (14), cardiovascular disease (16), and non-alcoholic fatty liver disease (26). Our study first reveals that in hypertensive patients, METS-VF also reliably predicts the risk of developing peripheral artery disease (PAD). Given that these patients often have elevated visceral fat and higher cardiovascular risk, this simple, low-cost tool can be easily incorporated into routine clinical care. Including METS-VF in regular check-ups allows earlier identification of high-risk individuals and enables personalized prevention. Such early intervention may help slow atherosclerosis progression, decrease PAD-related complications, and improve long-term health and quality of life in people with hypertension.

Several limitations of this study cannot be avoided. First, although multiple confounding factors were adjusted for in our analyses, the potential influence of unmeasured or residual confounding cannot be entirely ruled out. Second, our study participants were recruited exclusively from a rural hypertensive population in Southern China, which may limit the generalizability of our findings to other populations. Future research involving more diverse populations and geographic regions is warranted to validate the broad applicability of these results.

## Conclusion

In summary, this prospective cohort study demonstrated that a higher METS-VF was significantly associated with an increased risk of PAD among Chinese hypertensive patients, and a threshold effect of METS-VF with a cut-off point value of 8.63 on PAD.

## Data Availability

The raw data supporting the conclusions of this article will be made available by the authors, without undue reservation.
