# Supplementary material for: The metabolic score for visceral fat and risk of peripheral arterial disease in hypertension patients: a prospective cohort study
Source: Front Endocrinol (Lausanne). 2026 Feb 5;17:1764481. doi: 10.3389/fendo.2026.1764481 (PMC12916417; doi:10.3389/fendo.2026.1764481)
Supplement: Supplementary file 1 [file Table1.docx]

**Table S1 Association between MTES-VF and PAD in different models (unadjusted for BMI)**

| Exposure | Events, n (%) | PAD, HR(95%CI) | |
| --- | --- | --- | --- |
|  |  | Model 1 | Model 2 |
| Per 1 unit increase | 266 (4.12) | 1.11 (1.00, 1.23) | 1.11 (1.00, 1.24) |
| Tertiles |  |  |  |
| Q1(<7.30) | 87 (4.07) | 1.00 | 1.00 |
| Q2(≥7.31,<8.06) | 89 (4.12) | 1.20 (0.89, 1.63) | 1.23 (0.90, 1.68) |
| Q3(≥8.06) | 90 (4.18) | 1.46 (1.07, 2.00) | 1.49 (1.08, 2.06) |
| P for trend |  | 0.016 | 0.014 |

Model 1 was adjusted for age, sex

Model 2 was adjusted for age, sex, DBP, SBP, CHD, LDL-C, TC, eGFR, Hcy, Current smoking, Current drinking, Diabetes, Antihypertensive drugs, Lipoprotein-lowering drugs.

BMI, body mass index; SBP, systolic blood pressure; DBP, diastolic blood pressure; CHD, coronary heart disease; TC, total cholesterol; LDL-C, low density lipoprotein cholesterol; eGFR, estimated glomerular filtration rate; Hcy, homocysteine

**Table S2  Association between METS-VF and PAD in different models stratified by BMI of 24 kg/m^2^**

| MTES-VF | Events, n (%) | PAD, HR(95%CI) | |
| --- | --- | --- | --- |
|  |  | Model 1 | Model 2 |
| **BMI<24, kg/m^2^** |  |  |  |
| Tertiles |  |  |  |
| Q1(<7.30) | 85 (4.39) | 1.00 | 1.00 |
| Q2(≥7.31,<8.06) | 62 (5.69) | 1.30 (0.89, 1.90) | 1.14 (0.77, 1.69) |
| Q3(≥8.06) | 39 (6.82) | 1.83 (1.25, 2.67) | 1.64 (1.08, 2.49) |
| P for trend |  | <0.002 | 0.017 |
| **BMI≥24, kg/m^2^** |  |  |  |
| Tertiles |  |  |  |
| Q1(<7.30) | 2 (1.00) | 1.00 | 1.00 |
| Q2(≥7.31,<8.06) | 27 (2.52) | 1.93 (1.04, 3.59) | 2.14 (1.13, 4.06) |
| Q3(≥8.06) | 51 (3.23) | 2.01 (1.06, 3.78) | 2.66 (1.33, 5.35) |
| P for trend |  | 0.042 | 0.007 |

Model 1 was adjusted for age, sex

Model 2 was adjusted for age, sex, DBP, SBP, CHD, LDL-C, TC, eGFR, Hcy, Current smoking, Current drinking, Diabetes, Antihypertensive drugs, Lipoprotein-lowering drugs.

BMI, body mass index; SBP, systolic blood pressure; DBP, diastolic blood pressure; CHD, coronary heart disease; TC, total cholesterol; LDL-C, low density lipoprotein cholesterol; eGFR, estimated glomerular filtration rate; Hcy, homocysteine.
